# Consumer Perceptions and Acceptance of Edible Insects in Slovenia

**DOI:** 10.3390/foods13162629

**Published:** 2024-08-22

**Authors:** Nayyer Rehman, Nives Ogrinc

**Affiliations:** 1 WRG Europe Ltd., 26-28 Southernhay East, Exeter EX1 1NS, UK; nayyer@wrgeurope.co.uk; 2 Jožef Stefan International Postgraduate School, Jamova 39, 1000 Ljubljana, Slovenia; 3 Department of Environmental Sciences, Jožef Stefan Institute, Jamova 39, 1000 Ljubljana, Slovenia

**Keywords:** consumer perception, consumer acceptance, edible insects, sustainable consumption, food systems, Slovenia

## Abstract

Slovenia, influenced by Slavic, Mediterranean, and Balkan cultures, along with Austro-Hungarian traditions and strong environmental concerns, is an ideal case study for understanding consumer perceptions of edible insects and increasing their acceptance as an alternative protein source. A survey conducted in Slovenian and English with 537 participants examined existing perceptions and acceptance of edible insects as food and livestock feed. Findings show moderate interest in insects, particularly in non-visible, integrated forms, despite most participants not having tried whole insects. Young, educated individuals and those residing in rural areas have tried insects more often than other sociodemographic groups. Men showed more interest in entomophagy compared to women. Crickets, grasshoppers, and locusts were most acceptable, while cockroaches were least favored. Economic factors are crucial, with a preference for insect-based products priced similarly to conventional foods. The majority also support using insects as livestock feed. These results can aid policymakers at regional and national levels, help businesses market these products, and contribute to the literature on consumer responses in different European regions regarding edible insects as a sustainable food source.

## 1. Introduction

As the global population grows and traditional food systems face increasing pressures leading to protein shortage and environmental strain [1,2], the urgency to identify sustainable alternatives becomes critical. Since the 1970s, edible insects have emerged as a promising solution, attracting significant research interest, particularly in the last decade [3]. One reason is that insects are a highly nutritious food source [4], providing between 40 and 75 g/kg of digestible, high-quality protein [5,6], essential amino acids [7], fats and fiber [8]. Moreover, they contain bioactive compounds such as antioxidant enzymes and peptides [9], which may be beneficial in treating cancer and other diseases [10,11]. The low levels of investment and technical expertise required for insect farming make it a promising solution for combating global hunger, particularly among the most vulnerable populations [12], while also offering a more sustainable alternative for livestock [13,14] and aquaculture feed [15].

Insects, as a source of dietary protein [12,16,17], require considerably less land and water [18,19] than traditional livestock, needing only 3.6 m^2^ per kg of fresh insects (94.7 m^2^ on a dry weight basis) and between 0.42 and 0.82 m^3^ of water per kg of fresh insects [20]. They also generate fewer greenhouse gases, producing only 2.3 to 3.1 kg CO_2_ equivalents per kg of fresh insects [21]. Also, feeding insects on agricultural by-products and food waste could significantly reduce their price, a significant barrier to market entry [14,18,22,23,24], while helping mitigate food waste [25,26,27,28,29].

Despite their long history of widespread consumption in several countries [30,31], insects are met with significant skepticism, safety concerns, and unfamiliarity in Western societies [32,33,34,35]. Research indicates that the acceptability of insect-based foods is influenced by various factors, including sociodemographic characteristics, psychological factors, environmental awareness, knowledge and awareness, cultural norms, social influences and product features [36,37,38].

Sociodemographic characteristics such as gender, age, education level, and place of residence significantly impact consumer attitudes [37]. Men are generally more inclined to accept insects than women [39]. Younger individuals, especially those aged 20–40 and those with higher education levels, are also more open to entomophagy, while urban residents tend to be more accepting than those in rural areas [40].

Psychological factors also play a crucial role, with neophobia, disgust, and perceived health risks acting as substantial barriers; however, curiosity towards new food experiences can promote acceptability [41,42]. Positive attitudes, such as dietary habits and intentions to reduce meat consumption, also affect people’s willingness to try insect-based foods, although findings in this area are mixed [39,43].

Environmental awareness can positively influence the willingness to try insect-based products, though this effect is not always significant. While some studies suggest that sustainability concerns lead to higher acceptability, others find no considerable impact [38,39,43]. However, providing information on environmental benefits, especially before and after tasting sessions, can positively shift consumer perceptions [44,45].

Cultural compatibility and familiarity with insect consumption also play critical roles in shaping consumer attitudes. For example, previous exposure to entomophagy through knowledge or direct consumption increases acceptability, and positive tasting experiences, especially in social settings, can reduce disgust and food neophobia [46,47].

Finally, the characteristics of insect-based products—such as appearance, taste, visibility, and packaging—significantly influence consumer acceptance. Processed insect products are generally more acceptable than whole insects, and packaging that reduces insect visibility tends to be more favorably received. Additionally, product design, labeling, and pricing are crucial factors in shaping consumer preferences [48,49]. Understanding consumer attitudes is essential because their concerns can directly impact the success of a product [50,51,52]. Moreover, deeper insights into which sociodemographic groups are more likely to accept entomophagy allow for tailoring educational and marketing strategies that can effectively maximize resource efficiency and market impact [53,54].

While some consumers may respond positively to insect consumption due to its societal, environmental, and health benefits [54], others may require more direct sensory experiences to shift their perceptions [55,56]. Innovations such as using insect flour in baked goods promise to overcome food neophobia and enhance consumer openness to trying and incorporating insects into their diets [57,58,59,60,61].

While several studies have explored consumer acceptance of edible insects across Europe, there is limited understanding of how insect-based products are perceived in Slovenia. Adhering to European Union regulations for edible insects [62,63], Slovenia’s strategic location in Europe has resulted in a cultural heritage influenced by Slavic, Mediterranean, Balkan, and Austro-Hungarian traditions, which are also reflected in its food choices [64,65,66]. A recent survey by the European Investment Bank also revealed that Slovenia is one of only two countries in Europe that considers the environment the top challenge facing the nation [67]. These facts make Slovenia an excellent case study for examining how consumers in regions influenced by long-held traditions and cultures perceive and accept edible insects, an environmentally friendly alternative to traditional meat, and whether sociodemographic variables influence these perceptions.

This study provides the first comprehensive analysis of Slovenian consumer attitudes toward edible insects as food and feed through a questionnaire. Specifically, it examines how variables such as age, education, gender, and rural versus urban residency affect the acceptance of insects as a viable food source. The study also discusses variations in willingness to pay for these products and consumer attitudes toward products from animals fed on insects. Understanding these dynamics is crucial for policymakers and businesses venturing into edible insects to advance broader environmental objectives.

## 2. Materials and Methods

After reviewing the existing literature, we found no suitable survey tools in the Slovenian language for assessing the public perception of insect consumption and willingness to incorporate insects into their diets. To address this gap, we created a survey in Slovenian, which was then translated into English to make it accessible and inclusive for all residents. The survey questions were translated and reviewed by native speakers of both languages to maintain consistency and accuracy across translations.

### 2.1. Survey Design

The survey was created using Google Forms in June 2021. The survey design was based on the study by Kulma et al. [40] in the Czech Republic, comprising six primary questions focused on consumption habits, followed by demographic questions (Supplementary Material: Figures S1 and S2). Designed to be user-friendly, it included closed and multiple-choice questions and allowed respondents to skip sections if they were unfamiliar with entomophagy or chose not to answer.

The questionnaire was organized into three key sections: consumption habits and experiences, economic considerations, and demographic information. The consumption habits and experiences section assessed whether participants had tried eating insects and, if so, their experiences (positive, neutral, or negative) and their willingness to consume insect-based foods in the future. The economic considerations section focused on respondents’ price sensitivity, asking whether they would be willing to eat insects based on different pricing scenarios compared to conventional foods. It also gauged the acceptability of consuming various insect species in different forms, such as hidden in food, whole and raw, or whole and cooked, as well as their views on using insects as animal feed. Finally, the demographic information section gathered basic details such as age, gender, education level, and residence.

### 2.2. Data Collection

The questions were drafted and pre-tested with a small subset of respondents within the research group to ensure clarity and understandability, crucial for gathering meaningful data. The survey was distributed online through university networks and personal connections within the Jožef Stefan Institute and the Jožef Stefan Postgraduate School. Responses were collected between July and August 2021. Five hundred thirty-seven completed web questionnaires were received, with four hundred seventy-five in Slovenian and sixty-two in English.

To adhere to ethical guidelines applicable to surveys aimed at collecting public responses, this study implemented strict measures following the Personal Data Protection Act (ZVOP-1 [68], current version ZVOP-2 [69]) in Slovenia to ensure data confidentiality and anonymity. Before attempting the survey, respondents were informed about the study’s objectives, the voluntary nature of their involvement and the use of their responses in an academic context. No personal identifiers, such as names, email addresses, or IP addresses, were collected.

### 2.3. Statistical Analysis

The data analysis used the R statistical package (version 4.1.2). Descriptive statistics were employed to examine the sociodemographic variables of the participants and the consumption of various insects, which were visualized using a heatmap. We also performed several statistical tests to determine if there were statistical differences between the sociodemographic categories’ responses to questions related to the consumption of edible insects. For example, we used Chi-square tests to assess significant associations between categorical survey responses and sociodemographic variables (gender, age, residence, and education) and independence of observations to verify that all expected frequencies in the contingency tables were at least 5, fulfilling the conditions for valid Chi-square tests. For significant results (*p* < 0.05), we employed pairwise Chi-square tests with Bonferroni correction for each pair of categories within these variables to ensure that observed differences were robust and not due to chance from multiple testing. For significant associations, adjusted residuals were calculated, with values greater than ±2 indicating significant deviations from expected frequencies.

To examine the relationship between sociodemographic variables and individuals’ interest in tasting edible insects, we used an ordinal probit model. This model was chosen because it had lower Akaike Information Criterion (AIC) and Bayesian Information Criterion (BIC) values, indicating a better fit than the ordinal logit model. The response variable, which measured interest in tasting edible insects, was divided into four levels: ‘No’, ‘Somewhat no’, ‘Somewhat yes’, and ‘Yes’. The sociodemographic variables included were gender, age, education, and residence. We fitted the model and tested the significance of each variable using z-tests on the model coefficients, considering a *p*-value less than 0.05 as statistically significant.

## 3. Results

**Figure 1:**
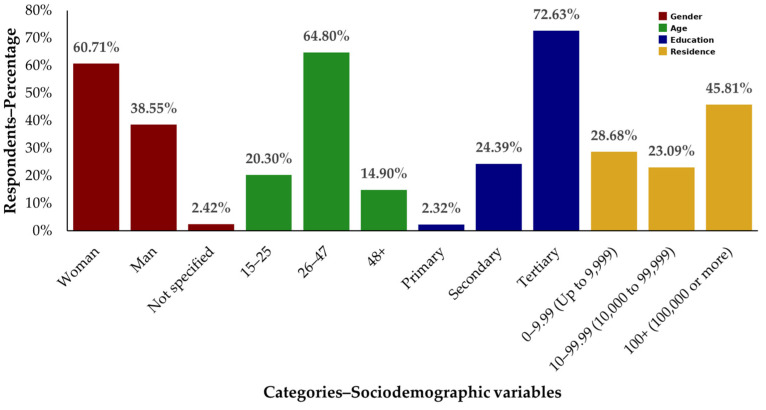
Demographic breakdown of respondents (%, *n* = 537).

The demographic breakdown of the 537 survey participants (Figure 1) shows that the majority were women (60.71%), followed by men (38.55%). Most respondents were aged 26–47 years (64.8%), with the next largest group being those aged 15–25 years (20.3%) and a smaller percentage over 48 years (14.9%). Regarding education, the majority of the respondents had tertiary education (72.62%), with secondary education as the next group (24.39%), with only a small fraction having primary education (2.23%). In terms of residence size, nearly half lived in areas with 100,000 inhabitants or more (45.81%), approximately a quarter in areas with up to 9999 inhabitants (28.68%), and the rest in areas with 10,000 to 99,999 inhabitants (23.09%).

Of the 537 respondents, only 96 (17.87%) reported having previous experience with eating insects, while the vast majority, 441 respondents (82.12%), had never tried them (Table 1). The results also showed that more men (19.32%) had eaten insects than women (16.56%). The Chi-square test showed no statistically significant difference across gender (χ^2^ = 5.75, *p* = 0.12). Younger respondents (15–25 years) were less likely to have eaten insects (9.17%) compared to those aged 26–47 years (21.84%) and those aged 48 and older (12.50%), with the Chi-square test indicating a statistically significant difference in insect consumption across age groups (χ^2^ = 10.90, *p* < 0.00). Individuals with primary education reported the highest percentage of having tried insects (33.33%), followed by those with tertiary education (19.23%) and secondary education (12.98%), although the Chi-square test showed no statistically significant difference across education levels (χ^2^ = 5.44, *p* = 0.14). The responses varied significantly with population size, with 25% of those living in areas with populations of 0–9.99 thousand and 27.27% of those in areas with populations of 10–99.99 thousand having tried insects, compared to 23.58% in larger populations (>100 thousand). The Chi-square test indicated a statistically significant difference in residence size (χ^2^ = 11.61, *p* = 0.01).

*Post hoc* comparisons indicated a significant difference between the age groups 26–47 and 15–25 (*p* = 0.01), while there were no significant differences between the age groups 26–47 and 48+ (*p* = 0.25), or between 15–25 and 48+ (*p* = 1.86). There was a significant difference between residents of areas with 0–9.99 thousand inhabitants and those with 10–99.99 thousand inhabitants (*p* < 0.00). No significant differences were found between residents of areas with 0–9.99 thousand inhabitants and those with more than 100 thousand inhabitants (*p* = 0.13) or between residents of areas with 10–99.99 thousand inhabitants and those with 100 thousand or more inhabitants (*p* = 0.22).

Among those who had eaten insects, experiences varied significantly. Positive experiences, i.e., with a willingness to eat insects again, were reported by 21.66% of respondents. Neutral experiences were reported by 20.83%, who would consider eating insects again under certain conditions, while 27.50% were neutral but preferred not to try insects again. Negative experiences were reported by 15.00%, who expressed no interest in eating insects again.

The survey also found varying responses based on sociodemographic variables (Table 2). Chi-square analysis revealed significant associations between responses to experiences of eating insects and the sociodemographic variables of age and residence. Age was significantly associated with responses to Q2 (χ^2^ = 10.76, *p* < 0.00). Younger respondents aged 15–25 reported a higher proportion of neutral experiences, with 35.71% not interested in trying insects again, and 28.57% possibly willing to try again, but no respondents in this age group reported positive experiences. Respondents aged 26–47 had a notable proportion (26.88%) interested in trying insects again, while those aged 48 and older showed the highest proportion of neutral, possibly willing to try again (46.15%). *Post hoc* comparisons indicated that the difference was significant between the age groups 26–47 and 15–25 (*p* = 0.01). No significant differences were observed between the other age groups.

A significant association was also observed between the experience of eating insects and the residence (χ^2^ = 12.69, *p* = 0.01). Individuals living in areas with populations of 0–9.99 thousand had the highest proportion of positive experiences (36.00%) and the lowest proportion of negative experiences (4.00%). In contrast, respondents from areas with populations of 10–99.99 thousand had the highest proportion of respondents choosing neutral, possibly willing to try again (30.77%). Those from areas with populations of 100 thousand or more had a higher proportion of negative experiences (18.84%) compared to other population sizes. After applying the Bonferroni correction, post hoc comparisons indicated no significant differences between the specific population categories.

For those who had not eaten insects, interest levels varied. No interest in trying edible insects was reported by 31.58%, 29.84% chose ‘Somewhat no’, 13.17% chose ‘Somewhat yes’, and 20.15% showed interest in trying edible insects in the future. The survey results indicated varying responses based on sociodemographic variables, as shown in Table 3.

A significant association was found between responses to Q3 and the sociodemographic gender (χ^2^ = 13.98, *p* < 0.00). Male respondents were more likely to express definite interest (32.83%) than female respondents (17.52%). The proportions of male and female respondents who were somewhat interested were almost the same; however, a higher proportion of female respondents (34.71%) were somewhat uninterested than male respondents (21.72%). In the case of a definite ‘No’, more women (33.76%) chose this option than men (28.79%). *Post hoc* analysis revealed that significantly more men chose ‘Yes’ (adjusted residual = 3.98), while significantly fewer women chose ‘Yes’ (adjusted residual = −3.98).

**Figure 2:**
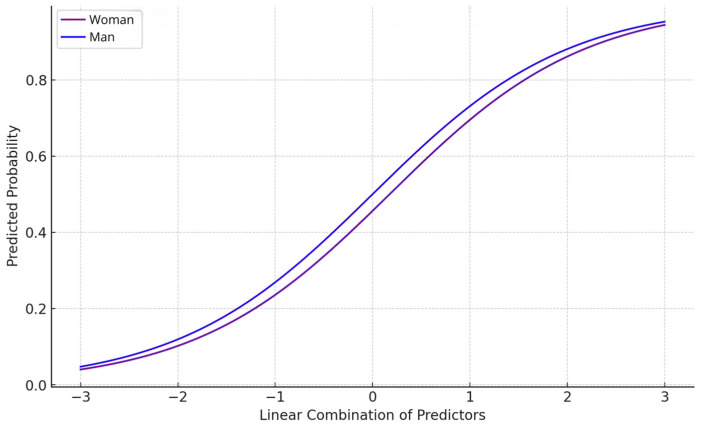
Predicted probabilities of interest levels by gender using the probit model.

The ordinal probit model analysis results identified gender as a significant predictor of interest (*β* = −0.1754, *p* = 0.001), indicating that men are more likely to express interest in tasting edible insects than women. In contrast, age (*β* = −0.1117, *p* = 0.207), education (*β* = −0.0714, *p* = 0.495), and residence (*β* = 0.0035, *p* = 0.954) were not statistically significant, suggesting these factors do not substantially influence interest. The model also estimated threshold values to mark points on the latent propensity scale where the probability of moving from one interest level to the next changes. The thresholds were −0.9234 (*p* < 0.001) between ‘No’ and ‘Somewhat no’, −0.2427 (*p* = 0.001) between ‘Somewhat no’ and ‘Somewhat yes’, and −0.9064 (*p* < 0.001) between ‘Somewhat yes’ and ‘Yes’. The significant negative coefficient for gender, combined with these thresholds, indicated that men are generally more likely to progress to higher levels of interest in tasting edible insects than women. Figure 2 shows the predicted probabilities: the blue line represents the predicted probabilities for men, while the purple line represents the predicted probabilities for women. The higher position of the blue line indicates that men have a higher likelihood of interest in tasting edible insects across the range of predictors.

**Figure 3:**
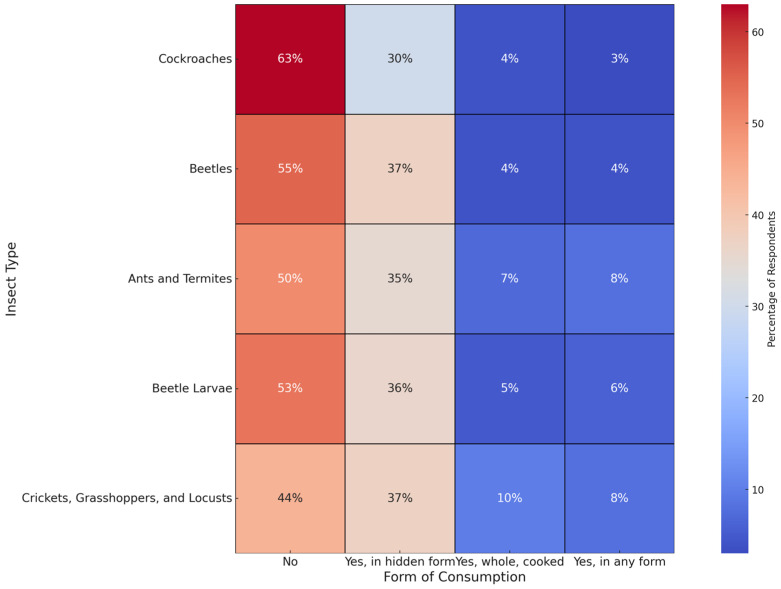
Heatmap of consumer acceptance for different insect types and forms of consumption.

In questions investigating the acceptability of consuming various insect types, the responses highlighted a significant preference for ingesting insects in non-visible, integrated forms (Figure 3). Crickets, grasshoppers, and locusts were the most favorably received, with 38.1% of participants open to consuming them in a hidden form, 9.1% accepting them whole and cooked, and 7.6% agreeable to consuming them in any form. This higher acceptance suggests these insects may not trigger disgust as others do, likely due to regional cultural and religious influences [65].

Ants and termites garnered a mixed reception, with 35.4% acceptance in hidden forms, 5.9% whole and cooked, and 7.4% in any form, reflecting perhaps a less pronounced bias due to their smaller size and less conspicuous nature. Beetle larvae and beetles also showed moderate acceptance levels; 36.0% and 36.8% of respondents, respectively, would consider these insects if disguised in food. Specifically, beetle larvae were acceptable to 4.2% of respondents in their whole form and 5.3% in any form, while beetles were acceptable to 3.6% whole and 3.4% in any form.

In contrast, cockroaches showed the lowest acceptability, with only 3.2% of respondents willing to consume them whole and cooked and 2.7% in any form, although 31.6% would consider them if presented in a hidden form. These results indicate strong cultural and psychological barriers against insect visibility in food.

From the 339 respondents who answered questions about the pricing of insects and insect-based foods, 173 (51.03%) indicated they would be willing to purchase such products if priced similarly to their conventional equivalents. Another 134 respondents (39.52%) stated they would consider incorporating insect-based products into their diet if the price was lower than that of conventional products. Only 32 respondents (9.43%) were prepared to buy insect-based products regardless of the higher price. This finding suggests significant price sensitivity among potential consumers of insect-based foods.

Regarding using insects as an alternative protein source for livestock, 325 respondents (60.52%) favored eating meat from livestock fed on insects, and 68 respondents (12.66%) were against it. Meanwhile, 144 respondents (26.81%) did not have an opinion on this topic, indicating some uncertainty or lack of knowledge about the implications of such practices.

## 4. Discussion

To the best of our knowledge, this survey provides the first online collected and statistically evaluated data on consumer perceptions toward insects in Slovenia, revealing crucial patterns that enhance our understanding of entomophagy in the region. A substantial proportion of Slovenian respondents who had never tried insects indicated a general reluctance or lack of opportunity to engage with this alternative protein source. Similar findings from Poland, Germany, Hungary, Switzerland, and the Czech Republic reflect consumer reluctance toward entomophagy as a result of cultural influences and limited exposure to insect-based products [70,71,72].

Approximately only 20% of respondents had tried insects, which could also indicate a limited market presence, with no commercial insect-based products currently available for most consumers to try. Among those who had tried insects, a notable proportion opted for negative or neutral responses and preferred not to try insects again, pointing to the necessity of addressing specific concerns related to neophobia, disgust, and sensory aspects (taste, texture, and presentation), as discussed in the literature [73,74,75,76,77].

Previous research identified sustainability as a key motivator for new consumers to try insect-based foods, particularly due to the reduced environmental impact and resource conservation associated with insect farming [11,19,37,75]. However, cultural and social norms remain significant barriers, especially in Western countries, where insect consumption is often associated with disgust and food neophobia. In contrast, in countries where entomophagy is already practiced, there is already an acceptance of insects as food, and the greater focus lies on the positive sensory attributes, availability, and affordability of insects [51,76].

Experiences of eating insects also varied significantly with age and education. The findings show that individuals in the 26–47 age group [15,34] and those with higher education levels [34,75] had more positive experiences with insects. This finding suggests that educational initiatives focusing on the nutritional and environmental benefits of entomophagy could be particularly effective in these demographics. Although individuals from rural areas had tried insects in a higher proportion than those residing in urban areas, there were no significant differences in their willingness to try insects in the future, possibly due to greater exposure and adoption of global food trends. The results also indicated that although there were no significant differences in gender for trying insects, a higher proportion of men were more likely to rate their experience positively and would try them again. These findings support previous researchers’ findings, who also found that male and young participants were more curious and adventurous regarding edible insects [32,39,40,61,78].

The findings also highlight varying degrees of acceptability towards insects and different forms of insect-based products, with the highest acceptance for crickets, grasshoppers, and mealworms, which is consistent with previous observations [79]. While overall willingness to consume insects in recognizable forms was low, a substantial proportion of respondents were open to consuming insects in hidden forms, such as powders and flour, which have shown positive consumer acceptance due to their taste and perceived higher nutritional value [40,45,80].

Price sensitivity emerged as a crucial factor influencing the potential consumption of insect-based products. More than half of the respondents indicated a willingness to purchase these products if priced similarly to equivalent conventional foods, while 40% preferred lower prices, and 10% were willing to pay more. This result emphasizes the need for competitively priced products as one of the strategies to increase acceptance [77].

Most survey respondents supported using insects as an alternative protein source for livestock, aligning with similar findings from a study by Verbeke [39], which noted favorable opinions among farmers, citizens, and agriculture sector stakeholders. Research also indicates acceptance of insect-based feeds in aquaculture across Mediterranean countries such as Spain, Greece, and Italy. Baldi et al. [81] noted that young Italian males and those aware of environmental benefits were more receptive to consuming insect-fed fish. Rumbos et al. [82] reported that most Greek participants at an aquaculture conference supported eating insect-fed fish, motivated by the potential to reduce pressure on wild fish stocks and enhance environmental sustainability.

Given that the findings show a moderate interest in eating insects among Slovenes, marketing efforts should focus on factors influencing consumers’ willingness to try insects [83]. Emphasizing positive sensory experiences and offering recipes can help consumers incorporate insects into their meals more easily. Barriers like disgust and fear of new foods are significant but can be reduced by presenting insects in processed forms, like flour or pastes, and incorporating them into familiar dishes [84,85].

Providing detailed information on the health and nutritional advantages, sustainability, safety, quality, and authenticity of edible insects can make them more appealing as a food choice [86]. These benefits can be highlighted through clear labeling, ingredient transparency, and information on sustainability parameters. Leveraging social media and celebrity endorsements could also position insect-based products as a sustainable alternative for receptive groups [87]. Most importantly, collaboration among government bodies, researchers, and businesses is essential to provide accurate information through educational programs [88], address concerns, and effectively promote the benefits of edible insects.

### Limitations and Future Research

Although the study has many strengths and provides insights into a country’s residents with diverse influences, it also has some limitations. The limited number of participants, mostly university students and staff, may not accurately represent the opinions of the wider Slovenian population. The study employs a quantitative approach with close-ended questions, which do not always capture the reasons behind participants’ choices in the survey. The questionnaire, adapted from the design of another study, covered important topics such as the price and use of insects for feed, but it did not address social reasoning, health and safety concerns, or individual perceptions of the environment. These aspects could have offered deeper insights and allowed for comparisons with other countries, extending the generalizability of the findings to other European nations with similar cultural and regional influences. Additionally, the study did not compare the costs of insect products with traditional foods.

Future research should consider including a larger sample size with a more diverse group, taking into account different ages and educational attainment levels to improve generalizability. The questionnaire should also include environmental concerns, health perceptions, and cultural dietary traditions for a more comprehensive understanding of consumer attitudes. Qualitative research methods could provide a deeper understanding of the rationale behind the responses to the questionnaire. Lastly, comparing insect products with comparable traditional foods and conducting a cost analysis could provide valuable information on consumer price sensitivity and preferences.

## 5. Conclusions

The findings from this study provide valuable insights into the sociodemographic factors influencing attitudes toward eating insects in Slovenia. Specific demographic groups, such as younger individuals, those with higher education, and men, could be targeted to increase the acceptance and integration of edible insects into mainstream diets. The data suggest that the most widely accepted way to consume insects in Slovenia is in hidden forms, such as insect flour. While market and cultural acceptance still require significant development, there is potential for insects as an alternative protein source. Addressing fears about taste and texture and communicating the benefits and safety of eating insects is crucial. Moreover, collaboration between businesses, researchers, and government will be vital to making insect-based foods a popular choice.

## Figures and Tables

**Table 1:** Associations between sociodemographic variables and trying insects (*n* = 537).

Q1: Have You Ever Tried Eating Insects?					
Sociodemographic Variables	Categories	No (%)	Yes (%)	Chi-Square (χ^2^)	*p*-Value
Gender	Man	80.68%	19.32%	5.75	0.12
	Woman	83.44%	16.56%		
	Not specified	33.33%	66.67%		
Age	15–25	90.83%	9.17%	10.90	<0.00
	26–47	78.16%	21.84%		
	48+	87.50%	12.50%		
Education	Primary education	66.67%	33.33%	5.44	0.14
	Secondary education	87.02%	12.98%		
	Tertiary education	80.77%	19.23%		
Residence	0–9.99	86.36%	13.64%	11.61	0.01
	10–99.99	86.29%	13.71%		
	100 and more	76.42%	23.58%		

**Table 2:** Associations between sociodemographic variables and experience with insects (*n* = 537).

Q2: Please Describe Your Experience with Insects								
Sociodemographic Variables	Categories	Negative, I Am Not Interested in Consuming Insects Anymore. (%)	Neutral, Rather Not Interested in Trying Again. (%)	Neutral, Possibly Will Try Again. (%)	Positive, Interested in Trying Insects Again. (%)	Positive, Regularly Consume Insects. (%)	Chi-Square (χ^2^)	*p*-Value
Gender	Man	17.65%	27.45%	13.73%	29.41%	0.00%	3.31	0.35
	Woman	13.43%	25.37%	26.87%	16.42%	1.49%		
	Not specified	0.00%	100.00%	0.00%	0.00%	0.00%		
Age	15–25	0.00%	35.71%	28.57%	0.00%	7.14%	10.76	<0.00
	26–47	17.20%	29.03%	16.13%	26.88%	0.00%		
	48+	15.38%	7.69%	46.15%	7.69%	0.00%		
Education	Primary education	0.00%	33.33%	33.33%	0.00%	33.33%	2.33	0.51
	Secondary education	8.00%	32.00%	24.00%	12.00%	0.00%		
	Tertiary education	17.39%	26.09%	19.57%	25.00%	0.00%		
Residence	0–9.99	4.00%	32.00%	12.00%	36.00%	4.00%	12.69	0.01
	10–99.99	15.38%	19.23%	30.77%	11.54%	0.00%		
	100 and more	18.84%	28.99%	20.29%	20.29%	0.00%		

**Table 3:** Associations between sociodemographic variables and interest in edible insects (*n* = 537).

Q3: Do You Have Any Interest to Taste Edible Insects in the Future?							
Sociodemographic Variables	Categories	No (%)	Somewhat No (%)	Somewhat Yes (%)	Yes (%)	Chi-Square (χ^2^)	*p*-Value
Gender	Man	28.79%	21.72%	13.64%	32.83%	13.98	<0.00
	Woman	33.76%	34.71%	13.06%	17.52%		
	Not specified	0.00%	50.00%	0.00%	50.00%		
Age	15–25	24.77%	38.53%	15.60%	21.10%	0.74	0.69
	26–47	34.14%	24.77%	13.29%	25.08%		
	48+	30.26%	39.47%	9.21%	19.74%		
Education	Primary education	8.33%	50.00%	25.00%	16.67%	4.47	0.22
	Secondary education	29.46%	34.88%	10.08%	24.03%		
	Tertiary education	32.61%	27.49%	14.02%	23.72%		
Residence	0–9.99	31.54%	31.54%	14.77%	20.81%	7.13	0.07
	10–99.99	25.42%	33.90%	13.56%	26.27%		
	100 and more	33.47%	26.27%	12.71%	24.58%		

## Data Availability

The original contributions presented in the study are included in the article/Supplementary Materials, further inquiries can be directed to the corresponding author.

## Supplementary Materials

The following supporting information can be downloaded at https://www.mdpi.com/article/10.3390/foods13162629/s1, Figure S1: Edible Insects Survey (Slovenian Version); Figure S2: Edible Insects Survey (English Version).

## References

[1] OECD/FAO (2020). OECD-FAO Agricultural Outlook 2020–2029.

[2] Godfray H.C.J., Beddington J.R., Crute I.R., Haddad L., Lawrence D., Muir J.F., Toulmin C. (2010). Food security: The challenge of feeding 9 billion people. Science.

[3] Meyer-Rochow V.B. (1975). Can insects help to ease the problem of world food shortage?. Search.

[4] Belluco S., Losasso C., Maggioletti M., Alonzi C.C., Paoletti M.G., Ricci A. (2013). Edible insects in a food safety and nutritional perspective: A critical review. Compr. Rev. Food Sci. Food Saf..

[5] Kouřimská L., Adámková A. (2016). Nutritional and sensory quality of edible insects. NFS J..

[6] Shah A.A., Totakul P., Matra M., Cherdthong A., Hanboonsong Y., Wanapat M. (2022). Nutritional composition of various insects and potential uses as alternative protein sources in animal diets. Anim. Biosci..

[7] Orkusz A. (2021). Edible Insects versus Meat—Nutritional Comparison: Knowledge of Their Composition Is the Key to Good Health. Nutrients.

[8] Tang C. (2019). Edible insects as a food source: A review. Food Prod. Process. Nutr..

[9] Zielińska E., Pankiewicz U., Sujka M. (2021). Nutritional, Physiochemical, and Biological Value of Muffins Enriched with Edible Insects Flour. Antioxidants.

[10] Wu R.A., Ding Q., Lu H., Tan H., Sun N., Wang K., He R., Luo L., Ma H., Li Z. (2020). Caspase 3-mediated cytotoxicity of mealworm larvae (*Tenebrio molitor*) oil extract against human hepatocellular carcinoma and colorectal adenocarcinoma. J. Ethnopharmacol..

[11] Ordoñez-Araque R., Egas-Montenegro E. (2021). Edible insects: A food alternative for the sustainable development of the planet. Int. J. Gastron. Food Sci..

[12] Van Huis A. (2021). Prospects of insects as food and feed. Org. Agric..

[13] Van Huis A., Gasco L. (2023). Insects as feed for livestock production. Science.

[14] Kuo C., Fisher B.L. (2022). A Literature Review of the Use of Weeds and Agricultural and Food Industry By-Products to Feed Farmed Crickets (Insecta; Orthoptera; Gryllidae). Front. Sustain. Food Syst..

[15] Sogari G., Oddon S.B., Gasco L., van Huis A., Spranghers T., Mancini S. (2023). Recent advances in insect-based feeds: From animal farming to the acceptance of consumers and stakeholders. Animal.

[16] Moruzzo R., Mancini S., Guidi A. (2021). Edible Insects and Sustainable Development Goals. Insects.

[17] Hamam M., D’Amico M., Di Vita G. (2024). Advances in the insect industry within a circular bioeconomy context: A research agenda. Environ. Sci. Eur..

[18] Dobermann D., Swift J.A., Field L.M. (2017). Opportunities and Hurdles of Edible Insects for Food and Feed. Nutr. Bull..

[19] Lange K.W., Nakamura Y. (2021). Edible Insects as Future Food: Chances and Challenges. J. Future Foods.

[20] Oonincx D.G.A.B., Van Broekhoven S., Van Huis A., Van Loon J.J. (2015). Feed Conversion, Survival and Development, and Composition of Four Insect Species on Diets Composed of Food By-Products. PLoS ONE.

[21] Oonincx D.G.A.B., Van Itterbeeck J., Heetkamp M.J.W., Van den Brand H., Van Loon J.J.A., Van Huis A. (2010). An exploration on greenhouse gas and ammonia production by insect species suitable for animal or human consumption. PLoS ONE.

[22] Varelas V. (2019). Food Wastes as a Potential New Source for Edible Insect Mass Production for Food and Feed: A Review. Fermentation.

[23] Ojha S., Bußler S., Schlüter O.K. (2020). Food Waste Valorisation and Circular Economy Concepts in Insect Production and Processing. Waste Manag..

[24] Mannaa M., Mansour A., Park I., Lee D.W., Seo Y.S. (2024). Insect-based agri-food waste valorization: Agricultural applications and roles of insect gut microbiota. Environ. Sci. Ecotechnol..

[25] Oonincx D.G.A.B., De Boer I.J.M. (2012). Environmental Impact of the Production of Mealworms as a Protein Source for Humans—A Life Cycle Assessment. PLoS ONE.

[26] Smetana S., Schmitt E., Mathys A. (2019). Sustainable use of Hermetia illucens insect biomass for feed and food: Attributional and consequential life cycle assessment. Resour. Conserv. Recycl..

[27] Van Broekhoven S., Oonincx D.G.A.B., Van Huis A., Van Loon J.J.A. (2015). Growth performance and feed conversion efficiency of three edible mealworm species (Coleoptera: Tenebrionidae) on diets composed of organic by-products. J. Insect Physiol..

[28] Čičková H., Newton G.L., Lacy R.C., Kozánek M. (2015). The use of fly larvae for organic waste treatment. Waste Manag..

[29] Imathiu S. (2020). Benefits and food safety concerns associated with consumption of edible insects. NFS J..

[30] Olivadese M., Dindo M.L. (2023). Edible Insects: A Historical and Cultural Perspective on Entomophagy with a Focus on Western Societies. Insects.

[31] Shockley M., Dossey A.T. (2014). Insects for Human Consumption. Mass Production of Beneficial Organisms.

[32] Kröger T., Dupont J., Büsing L., Fiebelkorn F. (2022). Acceptance of Insect-Based Food Products in Western Societies: A Systematic Review. Front. Nutr..

[33] Durst P.B., Johnson D.V., Leslie R.N., Shono K. (2010). Forest insects as food: Humans bite back. Proceedings of the a Workshop on Asia-Pacific Resources and their Potential for Development.

[34] Mancini S., Moruzzo R., Riccioli F., Paci G. (2019). European consumers’ readiness to adopt insects as food. A Review. Food Res. Int..

[35] Ribeiro J.C., Cunha L.M., Sousa-Pinto B., Fonseca J. (2018). Allergic risks of consuming edible insects: A systematic review. Mol. Nutr. Food Res..

[36] Mina G., Peira G., Bonadonna A. (2023). The Potential Future of Insects in the European Food System: A Systematic Review Based on the Consumer Point of View. Foods.

[37] Menozzi D., Sogari G., Veneziani M., Simoni E., Mora C. (2017). Eating Novel Foods: An Application of the Theory of Planned Behaviour to Predict the Consumption of an Insect-Based Product. Food Qual. Prefer..

[38] Hartmann C., Siegrist M. (2017). Consumer perception and behaviour regarding sustainable protein consumption: A systematic review. Trends Food Sci. Technol..

[39] Verbeke W. (2015). Profiling Consumers Who Are Ready to Adopt Insects as a Meat Substitute in a Western Society. Food Qual. Prefer..

[40] Kulma M., Tůmová V., Fialová A., Kouřimská L. (2020). Insect Consumption in the Czech Republic: What the Eye Does Not See, the Heart Does Not Grieve Over. J. Insects Food Feed.

[41] Ruby M.B., Rozin P. (2019). Disgust, sushi consumption, and other predictors of acceptance of insects as food by Americans and Indians. Food Qual. Prefer..

[42] Rozin P., Fallon A.E. (1987). A perspective on disgust. Psychol. Rev..

[43] Schösler H., de Boer J., Boersema J.J. (2012). Can we cut out the meat of the dish? Constructing consumer-oriented pathways towards meat substitution. Appetite.

[44] Looy H., Dunkel F.V., Wood J.R. (2014). How then shall we eat? Insect-eating attitudes and sustainable foodways. Agric. Hum. Values.

[45] Caparros Megido R., Gierts C., Blecker C., Brostaux Y., Haubruge É., Alabi T., Francis F. (2016). Consumer Acceptance of Insect-Based Alternative Meat Products in Western Countries. Food Qual. Prefer..

[46] Tan H.S.G., Fischer A.R.H., Tinchan P., Stieger M., Steenbekkers L.P., Van Trijp H.C. (2015). Insects as food: Exploring cultural exposure and individual experience as determinants of acceptance. Food Qual. Prefer..

[47] Berger S., Wyss A.M. (2020). The influence of social norms on the willingness to eat insects: A field experiment in Denmark and Switzerland. Appetite.

[48] Schouteten J.J., de Steur H., de Pelsmaeker S., Lagast S., Juvinal J.G., De Bourdeaudhuij I., Verbeke W., Gellynck X. (2016). Emotional and sensory profiling of insect-, plant- and meat-based burgers under blind, expected and informed conditions. Food Qual. Prefer..

[49] Tan H.S.G., van den Berg E., Stieger M. (2016). The influence of product preparation, familiarity, and individual traits on the consumer acceptance of insects as food. Food Qual. Prefer..

[50] House J. (2016). Consumer acceptance of insect-based foods in the Netherlands: Academic and commercial implications. Appetite.

[51] Hartmann C., Shi J., Giusto A., Siegrist M. (2015). The psychology of eating insects: A cross-cultural comparison between Germany and China. Food Qual. Prefer..

[52] Barsics F., Caparros Megido R., Brostaux Y., Barsics C., Blecker C., Haubruge E., Francis F. (2017). Could New Information Influence Attitudes to Foods Supplemented with Edible Insects?. Br. Food J..

[53] Kauppi S.M., Pettersen I.N., Boks C. (2019). Consumer Acceptance of Edible Insects and Design Interventions as Adoption Strategy. Int. J. Food Des..

[54] Batat W., Peter P. (2020). The Healthy and Sustainable Bugs Appetite: Factors Affecting Entomophagy Acceptance and Adoption in Western Food Cultures. J. Consum. Mark..

[55] Cunha L.M., Ribeiro J.C. (2019). Sensory and Consumer Perspectives on Edible Insects. Edible Insects in the Food Sector: Methods, Current Applications and Perspectives.

[56] Wendin K.M., Nyberg M.E. (2021). Factors Influencing Consumer Perception and Acceptability of Insect-Based Foods. Curr. Opin. Food Sci..

[57] Tan H.S.G., Tibboel C.J., Stieger M. (2017). Why Do Unusual Novel Foods Like Insects Lack Sensory Appeal? Investigating the Underlying Sensory Perceptions. Food Qual. Prefer..

[58] García-Segovia P., Igual M., Martínez-Monzó J. (2020). Physicochemical Properties and Consumer Acceptance of Bread Enriched with Alternative Proteins. Foods.

[59] Hartmann C., Siegrist M. (2016). Becoming an Insectivore: Results of an Experiment. Food Qual. Prefer..

[60] Orsi L., Voege L.L., Stranieri S. (2019). Eating edible insects as sustainable food? Exploring the determinants of consumer acceptance in Germany. Food Res. Int..

[61] Wilkinson K., Muhlhausler B., Motley C., Crump A., Bray H., Ankeny R. (2018). Australian consumers’ awareness and acceptance of insects as food. Insects.

[62] Żuk-Gołaszewska K., Gałęcki R., Obremski K., Smetana S., Figiel S., Gołaszewski J. (2022). Edible Insect Farming in the Context of the EU Regulations and Marketing—An Overview. Insects.

[63] Lotta F. (2019). Insects as Food: The Legal Framework. Edible Insects in the Food Sector: Methods, Current Applications and Perspectives.

[64] Hrnčič M.K., Cör D., Knez Ž. (2021). Food, Nutrition, and Health in Slovenia. Nutritional and Health Aspects of Food in the Balkans.

[65] Orkusz A., Orkusz M. (2024). Edible Insects in Slavic Culture: Between Tradition and Disgust. Insects.

[66] Milošević J., Žeželj I., Gorton M., Barjolle D. (2012). Understanding the Motives for Food Choice in Western Balkan Countries. Appetite.

[67] European Investment Bank Slovenes Believe Climate Change Is Now the Number One Challenge Facing the Country, EIB Survey Finds. https://www.eib.org/en/press/all/2023-464-slovenes-believe-climate-change-is-now-the-number-one-challenge-facing-the-country-eib-survey-finds#:~:text=Climate%20change%20impacts%20and%20environmental.

[68] National Assembly of the Republic of Slovenia Personal Data Protection Act (ZVOP-1). https://www.ip-rs.si/fileadmin/user_upload/doc/ZVOP-1_in_ZVOP-1a__English_/Personal_Data_Protection_Act_of_Slovenia_status_2013_final_eng.doc.

[69] National Assembly of the Republic of Slovenia Personal Data Protection Act (ZVOP-2). https://www.ip-rs.si/zakonodaja/zakon-o-varstvu-osebnih-podatkov/.

[70] Gere A., Székely G., Kovács S., Kókai Z., Sipos L. (2017). Readiness to Adopt Insects in Hungary: A Case Study. Food Qual. Prefer..

[71] Piha S., Pohjanheimo T., Lähteenmäki-Uutela A., Křečková Z., Otterbring T. (2018). The Effects of Consumer Knowledge on the Willingness to Buy Insect Food: An Exploratory Cross-Regional Study in Northern and Central Europe. Food Qual. Prefer..

[72] Zielińska E., Zieliński D., Karaś M., Jakubczyk A. (2020). Exploration of Consumer Acceptance of Insects as Food in Poland. J. Insects Food Feed.

[73] Mishyna M., Chen J., Benjamin O. (2020). Sensory Attributes of Edible Insects and Insect-Based Foods – Future Outlooks for Enhancing Consumer Appeal. Trends Food Sci. Technol..

[74] Mancini S., Sogari G., Menozzi D., Nuvoloni R., Torracca B., Moruzzo R., Paci G. (2019). Factors Predicting the Intention of Eating an Insect-Based Product. Foods.

[75] Onwezen M.C., Bouwman E.P., Reinders M.J., Dagevos H. (2020). A Systematic Review on Consumer Acceptance of Alternative Proteins: Pulses, Algae, Insects, Plant-Based Meat Alternatives, and Cultured Meat. Appetite.

[76] Florença S.G., Guiné R.P.F., Gonçalves F.J.A., Barroca M.J., Ferreira M., Costa C.A., Correia P.M.R., Cardoso A.P., Campos S., Anjos O. (2022). The Motivations for Consumption of Edible Insects: A Systematic Review. Foods.

[77] Van Huis A. (2020). Edible Insects. Handbook of Eating and Drinking.

[78] Schlup Y., Brunner T. (2018). Prospects for Insects as Food in Switzerland: A Tobit Regression. Food Qual. Prefer..

[79] Fischer A.R.H., Steenbekkers L.P.A. (2018). All Insects Are Equal, but Some Insects Are More Equal than Others. Br. Food J..

[80] Borges M.M., da Costa D.V., Trombete F.M., Câmara A.K.F.I. (2022). Edible Insects as a Sustainable Alternative to Food Products: An Insight into Quality Aspects of Reformulated Bakery and Meat Products. Curr. Opin. Food Sci..

[81] Baldi L., Mancuso T., Peri M., Gasco L., Trentinaglia M.T. (2021). Consumer Attitude and Acceptance toward Fish Fed with Insects: A Focus on the New Generations. J. Insects Food Feed.

[82] Rumbos C.I., Mente E., Karapanagiotidis I.T., Vlontzos G., Athanassiou C.G. (2021). Insect-Based Feed Ingredients for Aquaculture: A Case Study for Their Acceptance in Greece. Insects.

[83] van Huis A., Rumpold B. (2023). Strategies to Convince Consumers to Eat Insects? A Review. Food Qual. Prefer..

[84] Tan H.S.G., Verbaan Y.T., Stieger M. (2017). How Will Better Products Improve the Sensory-Liking and Willingness to Buy Insect-Based Foods?. Food Res. Int..

[85] Meyer-Rochow V.B., Hakko H. (2018). Can Edible Grasshoppers and Silkworm Pupae Be Tasted by Humans When Prevented to See and Smell These Insects?. J. Asia-Pac. Entomol..

[86] Rehman N., Edkins V., Ogrinc N. (2024). Is Sustainable Consumption a Sufficient Motivator for Consumers to Adopt Meat Alternatives? A Consumer Perspective on Plant-Based, Cell-Culture-Derived, and Insect-Based Alternatives. Foods.

[87] de Koning W., Dean D., Vriesekoop F., Aguiar L.K., Anderson M., Mongondry P., Oppong-Gyamfi M., Urbano B., Luciano C.A.G., Jiang B. (2020). Drivers and Inhibitors in the Acceptance of Meat Alternatives: The Case of Plant and Insect-Based Proteins. Foods.

[88] Gahukar R.T. (2016). Chapter 4—Edible Insects Farming: Efficiency and Impact on Family Livelihood, Food Security, and Environment Compared with Livestock and Crops. Insects as Sustainable Food Ingredients.

