# Supplementary material for: Consumer Perceptions and Acceptance of Edible Insects in Slovenia
Source: Foods. 2024 Aug 22;13(16):2629. doi: 10.3390/foods13162629 (PMC11353506; doi:10.3390/foods13162629)
Supplement: Supplementary file 1 [file foods-13-02629-s001.zip › foods-3161527-supplementary.pdf]

Figure S1: Edible Insects Survey (Slovenian Version)

Raziskava o stališčih in percepcijah do prehranjevanja z žuželkami v zahodnih kulturah

Entomofagija je izraz, ki jo uporabljamo v praksi uživanja žuželk kot hrane, zlasti pri ljudeh. Doslej je bilo zabeleženih več kot 2000 vrst žuželk, ki se uživajo v vseh oblikah in življenjskih obdobjih po vsem svetu v več kot 100 državah (Afrika, Azija, Oceanija in Južna Amerika). Užitne žuželke so hranljive in veljajo za človeka okolju prijazen vir beljakovin, vitaminov in aminokislin. Vseeno je uživanje žuželk v zahodni kulturi še vedno tabu, a čeprav so užitne žuželke le redko na jedilniku, je prehod na prehranjevanje žuželk široko obravnavana tema, saj se članki v medijih pojavljajo skoraj vsak dan. Evropska komisija je že prejela številne prijave novih živil, povezanih z žuželkami. Od tistih žuželk, ki so vstopile v fazo ocene tveganja, je EFSA odobrila suhe rumene mokarje (ličinke Tenebrio molitor) za prehrano ljudi in bi jih lahko v skladu z Uredbo (EU) 2015/2283 šteli za novo živilo. Nekatere evropske države pravzaprav že dovoljujejo prodajo žuželk kot hrano v skladu s „prehodnimi ukrepi“ v skladu z novo zakonodajo o hrani. Kljub temu še vedno ni jasno, ali to odraža pomemben premik zahodnega odnosa do žuželk kot alternativnega vira hrane, kar predstavlja osnovo za nadaljnje raziskave.

Ta vprašalnik je bil oblikovan v znanstvene namene v sodelovanju Univerze za življenjske znanosti v Pragi, Češka z Institutom "Jožef Stefan". Glavni namen je raziskati stališča in percepcije do prehranjevanja žuželk v zahodnih kulturah. Izpolnjevanje vprašalnika vam bo vzelo približno 5 minut vašega časa. Rezultati ankete so namenjeni samo akademskim raziskavam (prostovoljno sodelovanje), vsi odgovori pa bodo anonimni.

Za vaš čas in sodelovanje se vam najlepše zahvaljujemo!

1. Ali ste kdaj uživali žuželke?
   - O Da. Preskočite na vprašanje 2.
   - O Ne. Preskočite na vprašanje 3.
2. Prosim, opišite vašo izkušnjo pri uživanju žuželk.
   - O Pozitivno, redno uživam žuželke.
   - O Pozitivno, ponovno bi jedel žuželke.
   - O Nevtralno, bi ponovil in če bi mi bilo všeč, bi jih jedel.
   - O Nevtralno, morda bi ponovno jedel žuželke.
   - O Negativno, uživanje žuželk me ne zanima več. Preskočite na vprašanje 6.
   - O Ostalo: Preskočite na vprašanje 4.
3. Ali bi želeli v prihodnosti okusiti užitne žuželke?
   - O Da.
   - O Bolj da kot ne.
   - O Ne. Preskočite na vprašanje 6.
   - O Raje ne.
   - O Ostalo: Preskočite na vprašanje 4.
4. S cenovnega vidika, ali ste pripravljeni jesti žuželke?
   - O Le če bi bila cena nižja od običajnih živil.
   - O Če bi bila cena primerljiva z običajnimi živili.
   - O Tudi če bi bila cena višja od običajnih živil.
5. Izberite sprejemljivost uživanja naslednjih skupin vrst:

	Da, v katerikoli obliki	Da, v skriti obliki	Da, cele in surove	Da, cele in kuhane	Ne
Ščurki	O	O	O	O	O
Hrošči	O	O	O	O	O
Mravlje in termiti	O	O	O	O	O
Ličinke hroščev	O	O	O	O	O
Črički, cvrčalke in kobilice	O	O	O	O	O

6. Kakšno je vaše mnenje o krmljenju živine z žuželkami? Bi uživali meso živine, ki se hrani z žuželkami?
   - O Pozitivno – da, bi.
   - O Negativno – ne, ne bi.
   - O Nimam mnenja o tej temi.
7. Spol
   - O Ženska
   - O Moški
   - O Nedoločeno
8. Starost
   - O 15-25
   - O 26-47
   - O 48+
9. Izobrazba
   - O Primarna izobrazba
   - O Sekundarna izobrazba
   - O Terciarna izobrazba
10. Prebivališče (v tisočih prebivalcev)
    - O 0-9,99
    - O 10-99,99
    - O 100 and more

Figure S2: Edible Insects Survey (English Version)

Attitudes and Perceptions Towards Eating Insects in Western Cultures

Entomophagy refers to eating insects as food, especially by people. At present, more than 2000 insect species are eaten in more than 100 countries (Africa, Asia, Oceania and South America) in all varieties and life stages. Edible insects are nutritious and considered an environmentally sustainable source of protein, vitamins, and amino acids for humans. All the same, insect consumption is still taboo in Western culture, but although edible insects are rarely on the menu, the move towards eating insects is a widely discussed topic, with articles appearing in the media almost daily. The European Commission has already received many insect-related novel food applications. Of those insects that have entered the risk assessment phase, dried yellow mealworms (Tenebrio Molitor larva) have been approved for human consumption by the EFSA and could be considered a novel food pursuant to Regulation (EU) 2015/2283. In fact, some European countries have already permitted the sale of insects as food under the "transition measures" of the Novel Food law. However, it remains to be investigated whether this reflects a significant shift in Western attitudes towards eating insects.

This questionnaire was created solely for scientific purposes by researchers at the Czech University of Life Sciences, Prague and Slovenia's Jožef Stefan Institute, Ljubljana, to explore Western people's attitudes and perceptions towards eating insects. The completion of the questionnaire will take approximately 5 minutes. The survey results are for academic research only (voluntary participation), and all answers will be kept anonymous.

We sincerely thank you for your time and participation!

1. Have you ever tried eating insects?
   - O Yes (Skip to Question 2)
   - O No (Skip to Question 3)
2. Please describe your experience of eating insects.
   - O Positive, I am eating insects regularly
   - O Positive, I am interested in trying insects again
   - O Neutral, rather not interested in trying again
   - O Neutral, possibly will try again
   - O Negative: I am not interested in consuming insects anymore (Skip to Question 6)
   - O Others: Skip to Question 4
3. Do you have any interest in tasting edible insects in the future?
   - O Yes
   - O Somewhat yes
   - O No (Skip to Question 6)
   - O Somewhat no
   - O Others: Skip to Question 4
4. From the point of pricing, you are willing to eat insects:
   - O Only if the price would be lower than conventional foods
   - O If the price would be comparable to conventional foods
   - O Even if the price would be higher than conventional foods
5. Choose an acceptability of consumption of the following species groups:

	Yes, in any form	Yes, in hidden form	Yes, whole, raw	Yes, whole, cooked	No
Cockroaches	O	O	O	O	O
Beetles and bugs	O	O	O	O	O
Ants and termites	O	O	O	O	O
Beetle larvae	O	O	O	O	O
Crickets, katydids and locusts	O	O	O	O	O

6. What is your opinion about feeding livestock with insects? Would you consume meat from livestock fed on insects?
   - O Positive, yes, I would
   - O Negative, no, I would not
   - O I do not have an opinion on this topic
7. Gender
   - O Woman
   - O Man
   - O Not specified
8. Age
   - O 15-25
   - O 26-47
   - O 48+
9. Education
   - O Primary education
   - O Secondary education
   - O Tertiary education
10. Residence (in thousands of inhabitants)
    - O 0-9.99
    - O 10-99.99
    - O 100 and more
